# *aes*, the gene encoding the esterase B in *Escherichia coli*, is a powerful phylogenetic marker of the species

**DOI:** 10.1186/1471-2180-9-273

**Published:** 2009-12-29

**Authors:** Mathilde Lescat, Claire Hoede, Olivier Clermont, Louis Garry, Pierre Darlu, Pierre Tuffery, Erick Denamur, Bertrand Picard

**Affiliations:** 1 INSERM U722 and Université Paris 7, Paris, France; 2 INSERM U535, Villejuif, France; 3 INSERM U436 and Université Paris 7, Paris, France; 4 INSERM U722 and Université Paris 13, Paris, France

## Abstract

**Background:**

Previous studies have established a correlation between electrophoretic polymorphism of esterase B, and virulence and phylogeny of *Escherichia coli*. Strains belonging to the phylogenetic group B2 are more frequently implicated in extraintestinal infections and include esterase B_2 _variants, whereas phylogenetic groups A, B1 and D contain less virulent strains and include esterase B_1 _variants. We investigated esterase B as a marker of phylogeny and/or virulence, in a thorough analysis of the esterase B-encoding gene.

**Results:**

We identified the gene encoding esterase B as the acetyl-esterase gene (*aes*) using gene disruption. The analysis of *aes *nucleotide sequences in a panel of 78 reference strains, including the *E. coli *reference (ECOR) strains, demonstrated that the gene is under purifying selection. The phylogenetic tree reconstructed from *aes *sequences showed a strong correlation with the species phylogenetic history, based on multi-locus sequence typing using six housekeeping genes. The unambiguous distinction between variants B_1 _and B_2 _by electrophoresis was consistent with Aes amino-acid sequence analysis and protein modelling, which showed that substituted amino acids in the two esterase B variants occurred mostly at different sites on the protein surface. Studies in an experimental mouse model of septicaemia using mutant strains did not reveal a direct link between *aes *and extraintestinal virulence. Moreover, we did not find any genes in the chromosomal region of *aes *to be associated with virulence.

**Conclusion:**

Our findings suggest that *aes *does not play a direct role in the virulence of *E. coli *extraintestinal infection. However, this gene acts as a powerful marker of phylogeny, illustrating the extensive divergence of B2 phylogenetic group strains from the rest of the species.

## Background

In humans, *Escherichia coli *strains can be commensal (part of the normal intestinal microbiota) and/or the cause of various infectious diseases (intestinal and extraintestinal infections) [1]. The extent of commensal or virulent properties displayed by a strain is determined by a complex balance between the status of the host and the production of virulence factors in the bacteria. The role of the intrinsic virulence of the isolates needs to be clarified and molecular markers of virulence are required to predict the invasiveness of clinical strains isolated during the course of extraintestinal infection or patient colonization.

*E. coli *has a clonal genetic structure and exhibits a low level of recombination [2]. *E. coli *strains can be categorised into four main phylogenetic groups, A, B1, B2, and D. These groups have been defined based on proteic (multi-locus enzyme electrophoresis including the electrophoresis of esterases [3]) and genetic markers (restriction fragment length polymorphism [4], random amplified polymorphic DNA [4] and multi-locus sequence typing (MLST) [5,6]). Seven types of esterases (A, B, C, D, I, F and S), differing in their ability to hydrolyse synthetic substrates and their sensitivity to di-isopropyl fluorophosphate, have been identified by separation on polyacrylamide agarose gels [7-9]. The most frequently observed type in this group of enzymes corresponds to esterase B (EC 3.1.1.1). This protein shows two types of electrophoretic mobility: B_1 _from M_f _= 74 to M_f _= 66 and B_2 _from M_f _= 63 to M_f _= 57 [9]. Strains with type B_2 _esterase belong to the phylogenetic group B2, whereas those with type B_1 _esterase belong to the non-B_2 _phylogenetic groups [10]. Several studies have shown a correlation between long-term evolutionary history (strain phylogeny) and virulence in *E*. *coli*, with most extraintestinal *E*. *coli *pathogens (including urinary tract infection strains) belonging to just one of the four main *E*. *coli *phylogenetic groups, the phylogenetic group B2 [11-13]. This correlation suggests a possible link between esterase polymorphism and extraintestinal virulence in an asexual species with a low level of recombination. Esterase B allozymes therefore appear to act as efficient molecular markers of virulence dividing *E. coli *strains into two genetically distinct groups, which differ significantly in their pathogenicity. However, the direct role of esterase B, or of its B_1 _and/or B_2 _allozymes, in the virulence process remains unknown.

The aims of this study were (i) to identify the gene encoding esterase B, (ii) to analyse its polymorphic counterparts in relation to *E. coli *clonal structure, (iii) to identify a potential physical link between this genetic locus and regions known to be associated with pathogenicity in the *E. coli *genome, and (iv) to test a potential direct role of esterase B in virulence in a mouse model of extraintestinal infection.

## Results and Discussion

### The acetyl esterase gene (*aes*) encodes esterase B

Seven candidate genes encoding proteins with predicted esterase activity were identified, based on their respective PM and pI values, using the MaGe system [14] (*aes *[15], *yddV*, *glpQ, ndk, yzzH *and *cpdA*). Of these, Aes exhibited several characteristics particularly reminiscent of esterase B: i) a major esterase domain, ii) a theoretical pI of 4.72 for the K-12 strain protein (esterase B_1_, pI ranging from 4.5 to 4.8) and 5.18 for CFT073 protein (esterase B_2_, pI ranging from 4.85 to 5.0), and iii) the presence of a serine in the active site [9]. The inactivation of *aes *by gene disruption in K-12 MG1655 and CFT073 strains and complementation of the mutant strains with the *aes *gene confirmed that Aes was esterase B (Additional file 1: Fig. S1 and data not shown).

**Figure 1:**
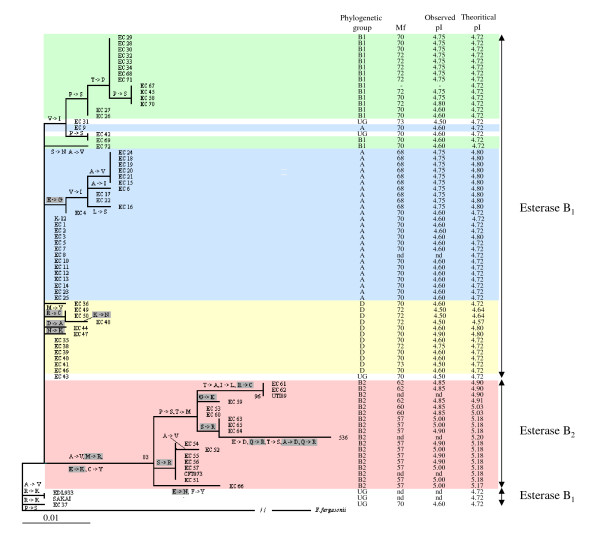
**Phylogenetic tree of Aes sequences from the 72 ECOR strains and 6 *E. coli *reference strains**. The tree was reconstructed with PHYML [50]. *E. fergusonii *was used as an outgroup. Bootstraps are shown for values higher than 70%. Differences in amino acids are indicated on the branches. Differences for each branch were derived from comparison of consensus amino-acid sequences of the ancestors and descendants. Boxed amino-acid substitutions correspond to substitutions that change the overall pI of the protein. The phylogenetic groups A (blue box), B1 (green box), B2 (red box), D (yellow box) and ungrouped strains (UG) (white box), electrophoretic mobilities (Mf) obtained by polyacrylamide agarose gel electrophoresis [10] and the observed [10] and theoretical pI of Aes are indicated. nd: non determined. -: non significant results.

We then studied the correlation between Aes sequences and esterase B electrophoretic polymorphism. The comparison of the Aes phylogenetic tree with the theoretical and observed pI values and the esterase B electrophoretic mobilities (Mf values) for the 72 ECOR strains [10] is shown in Fig. 1. Overall analysis of the tree confirmed separation of esterase B into two variants: esterase B_1 _and esterase B_2_. Indeed, the Aes tree showed a clear distinction between Aes from the phylogenetic group B2 strains and Aes proteins from other strains, separated by a long branch, well supported by bootstrap (83%). Moreover, the characterisation of the phylogenetic group B2, based on Aes polymorphism, was consistent with the pI and Mf values of esterase B_2 _(pI: 4.85 to 5.0 and Mf 57 to Mf 62), which were previously demonstrated to be specific to the phylogenetic group B2. Likewise, the characterisation of the phylogenetic groups A, B1 and D, based on Aes polymorphism, correlated with the pI and Mf values of esterase B_1 _(pI: 4.60 to 4.80 and Mf 68 to Mf 72) [10]. Amino-acid substitutions detected from the branches of the Aes tree were analysed taking into account variation in esterase B mobility and pI values [16] (Fig. 1). In most cases, for the Aes phylogenetic group B2 strains, substitutions of acidic to neutral, neutral to basic or acidic to basic amino acids corresponded to increases in pI (from 4.85 to 5.00) and decreases in Mf values (from 62 to 57) among esterase B variants. However, there were some discrepancies. For example, the substitution of a basic amino acid in the ECOR 53 and 60 strains by a neutral amino acid in the ECOR 61 and 62 strains (R?C) corresponded to a faster migration in the ECOR 61 and 62 strains (Mf values 62 versus 60), with no effect on pI (4.85) (Fig. 1).

A more complex pattern of polymorphism was found among the A, B1 and D phylogenetic group strains. Taking the most frequent esterase B electrophoretic variant (pI: 4.60 and Mf 70) detected in the phylogenetic group A and D strains, an acidic to neutral amino-acid change (E?G) led to an increase in pI (from 4.60 to 4.75) and a decrease of Mf (from 70 to 68) of the esterase B variant, as expected. This amino-acid change was detected in 11 strains in the phylogenetic group A (Fig. 1). In contrast, several discrepancies were found among strains belonging to the phylogenetic B1 group: Aes polymorphism included several substitutions of neutral to neutral amino acids but with increased pI values (from 4.60 to 4.75) and in some cases paradoxical increases of Mf values (from 70 to 72) was observed (Fig. 1). These apparent discrepancies may be due to the effects of conformational or post-translational modifications of the protein.

### The phylogenetic history of *aes *reflects the species phylogeny

To determine the evolutionary history of *aes*, we tested for selection using the *aes *sequence from 78 studied strains. First, we used a one-ratio model (M0) to estimate the average ratio ω (dN/dS) for all sites and all lineages at 0.18. The likelihood ratio test suggested that *aes *was under strong global purifying selection (compared to the neutral hypothesis which is ω = 0). The M1a, M2a, M7 and M8 models, estimating the selection on specific codons, confirmed that the vast majority (91%) of the sites are under negative selection. Finally, the branch-site model A did not detect positive selection along the branch separating group B2 from group non-B2 strains. Thus, the use of several tests for selection has shown that the evolution of *aes *has been driven essentially by purifying selection, as observed for the housekeeping genes used in the MLST (data not shown); no positively selected site was identified for the branch separating esterase B_2 _from esterase B_1 _strains. Previous studies based on whole genome sequencing data using PAML have not identified *aes *to be under positive selection [17,18].

**Figure 2:**
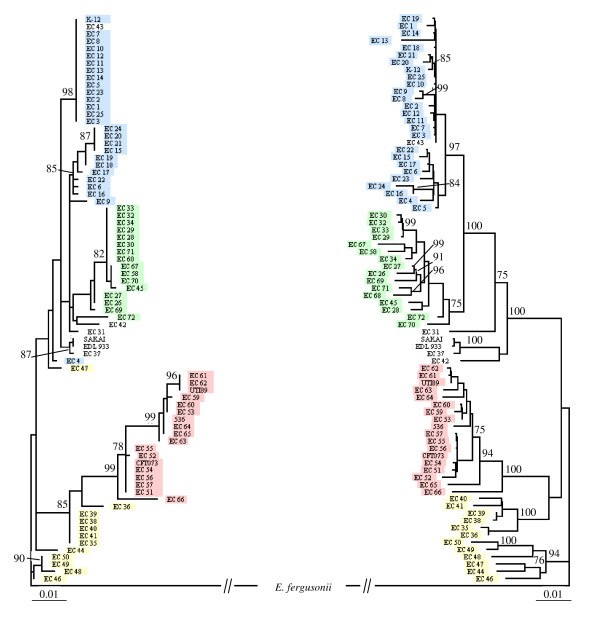
**Phylogenetic trees for the 72 ECOR strains and six *E. coli *reference strains**. The trees were constructed from (A) *aes *sequences and (B) multi-locus sequence typing of six housekeeping genes representing the species phylogeny (*trpA*, *trpB*, *pabB*, *putP*, *icd *and *polB*) [5], obtained using PHYML procedure [50]. *E. fergusonii *was used as an outgroup. Bootstraps are shown for values higher than 70%. Strains studied and belonging to phylogenetic groups A (blue boxed), B1 (green boxed), B2 (red boxed), D (yellow boxed) and UG (white boxed) are indicated.

Visual comparison of the phylogenetic history of *aes *with that of the six concatenated housekeeping genes, reflecting the species phylogeny, revealed a similar topology with four main phylogenetic groups (Fig. 2). Indeed, all strains belonging to the B2 phylogenetic group were clustered in a monophyletic group (bootstrap 99%) with ECOR 66 at its base, as observed in the MLST tree. Likewise, two sub-groups were observed for phylogenetic group D, one of which was associated with the phylogenetic group B2 (ECOR 35, 36, 38, 39, 40, 41) (bootstrap 85%), also observed in the MLST tree. Phylogenetic group A also constituted two sub-groups, although these were not sister groups. By contrast, the B1 phylogenetic group was monophyletic overall, with only two strains (ECOR 4 and ECOR 47) clearly misclassified (Fig. 2).

**Figure 3:**
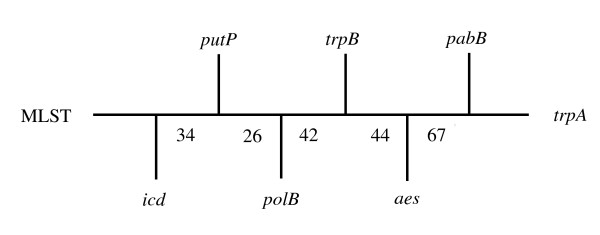
**Tree representing the distance matrix generated from comparisons between gene tree structures**. Gene tree structure comparisons were between trees based on *aes *sequences, six individual housekeeping genes (*trpA*, *trpB*, *pabB*, *putP*, *icd *and *polB*) and multi-locus sequence typing (concatenation of the six housekeeping genes), with distances derived from path-length difference. Numbers are bootstraps.

We used a recently developed technique ("TreeOfTree") allowing the level of congruence between phylogenetic trees to be tested [19]. We tested each individual housekeeping gene tree, the MLST tree, and the *aes *tree. All the bootstraps are low enough (less than 67%) to suggest that all the gene trees can be view as not incongruent, the *aes *gene tree itself clustering with *pabB *and *trpA *gene trees with very low bootstrap (44%) (Fig. 3). Thus, *aes *tree topology showed that *aes *is a powerful marker of the species phylogeny, as observed for each housekeeping gene used in the MLST scheme.

**Figure 4:**
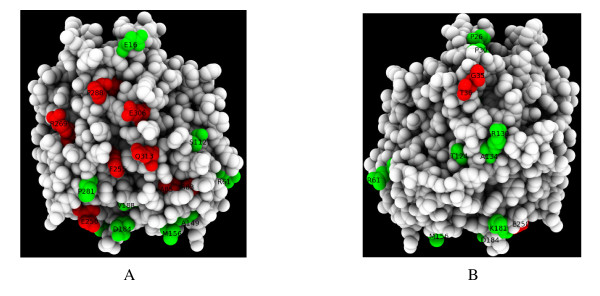
**Models of the Aes protein variants**. Of the 38 polymorphic sites identified, only the 24 sites at the protein surface are represented. Polymorphic sites are in green for carboxylesterase type B_1 _and red for type B_2_. The views A and B correspond to two opposite faces of the structure obtained by a rotation of 180° around the Y axis. Images were generated using PMG [57].

Aes B_1 _and B_2 _protein variants were then compared by protein modelling. We found that residues S 157, D 254 and H 284 had a geometry similar to that of the esterase catalytic site. Thirty-eight polymorphic sites were identified by comparing the 319 Aes amino-acid sequences obtained for the 72 ECOR strains and six reference strains. For four of these sites, variation has become fixed in both B_1 _and B_2 _types, with the identified residues differing between the two types at each site. These polymorphisms could thus be used to distinguish between the types: the B_1 _conserved amino acids A 53, M 64, E 73 and C 78 correspond to the B_2 _conserved amino acids V, R, K and Y, respectively. These four polymorphic sites were found on the long B2/non B2 branch in the proteic tree, explaining the observed high bootstrap (83%) (Fig. 1). Fig. 4 shows the location of 24 additional sites at the protein surface with observed amino-acid variants for either type B_1 _(green) or type B_2 _(red). No one site was polymorphic for both B_1 _and B_2 _types. But for all the polymorphic sites within types B_1 _and B_2_, some of the amino-acid variants are shared by the two types. Consequently, these sites cannot be considered to be specific to either one type or the other and cannot be used to distinguish between the two types of protein. Polymorphic sites were clustered, localised at the surface and were not found in the active site, consistent with previous observations of similarity in the catalytic activity of B_1 _and B_2 _esterases with synthetic substrates [7,9]. These differences in location of the polymorphic sites between the two variants support the divergence of the B2 phylogenetic group strains from the A, B1 and D phylogenetic groups strains within this species.

### Is Aes involved in virulence?

The previously observed correlation between electrophoretic esterase B polymorphism and the distinction between B2 and non-B2 phylogenetic group strains [10] - and thus with the extraintestinal virulence of the strains - suggested a putative role for the enzyme, or certain variants, as a virulence factor. The esterase B hydrolase function may have a direct role in the colonization or invasion of the eukaryotic cells as it was observed for esterases in other bacteria [20,21]. Indeed, esterase B_2 _variants belonging to phylogenetic group B2 may confer higher levels of virulence to the strain during extraintestinal infection. There are several examples of proteins with variants playing different roles in extraintestinal infections: the adhesins FimH [22], PapG [23] and the somatic antigen O [24,25].

Previous studies of Aes have not demonstrated a role of the protein in virulence. Firstly, experimental studies characterising Aes as an enzyme with esterase activity have demonstrated the inhibitory interaction of Aes with MalT, a transcriptional regulator of the maltose regulon. These findings suggested a role for Aes in the regulation of maltose metabolism [15,26]. Aes may also play a role in the regulation of raffinose metabolism by inhibiting α-galactosidase [27]. However, these data were obtained from overexpression of *aes *from plasmids, thus raising the question of their relevance *in vivo*. An illustration of *aes *overexpression from the plasmid pACS2 [28] is shown in Additional file 1: Fig. S1. Secondly, a previous study of *aes *expression in the K-12 strain *in vitro *did not find significant effects on expression under the various metabolic, stress or environmental conditions tested http://genexpdb.ou.edu/, with the exception of *aes *overexpression observed in strains cultured in the presence of acetate [29]. Interestingly, esterase B exhibits Michaelis-Menten kinetics for the hydrolysis of 1-naphtyl acetate [9]. Finally, *aes *expression was found to be homogeneous across 10 representative strains of *E. coli/Shigella *cultured in 869 medium [30].

Our previous findings from the study of the genetic sequence surrounding *aes *did not suggest a role for the encoded protein in virulence. Indeed, comparisons, using the MaGe system, of 75 kbp of sequence upstream and downstream from *aes *in the 20 strains of *E. coli *[31] showed that *aes *is not located in/or adjacent to any regions linked to extraintestinal pathogenicity specific to B2 strains (Additional file 2: Table S1).

To gain insight into Aes function we tested the mutants under different conditions. Firstly, we studied the *in vitro *growth of parent-type strains and their respective mutants on several carbon sources. We did not observe any difference between parent-type strains K-12 or CFT073 and their respective mutants K-12 Δ*aes *and CFT073 Δ*aes *in competition studies with LB and gluconate minimum media (data not shown). Additionally, growth of the strains CFT073, K-12, CFT073 Δ*aes *and K-12 Δ*aes*, in the presence of different carbon sources, was the same for parent and mutant strains. These results suggested that Aes does not play a role in regulation of the growth of the strains in these conditions. Secondly, we studied whether Aes is involved in the virulence of *E. coli in vivo *using a septicaemia mouse model. Kaplan-Meyer curves obtained for CFT073 and its mutants CFT073 Δ*aes *and CFT073 Δ*aes*:Cm were similar, suggesting that Aes is not involved in the virulence process (p = 0.87) (Additional file 1: Fig. S2).

## Conclusion

Selection tests and phylogenetic analyses indicate that *aes *is under purifying selection, showing a similar evolutionary history to that of the species. The differences in electrophoretic properties between the variant types B_1 _and B_2 _were consistent with analyses of the amino-acid sequence tree for Aes and protein structure models obtained for these variants. These findings illustrated the marked divergence of the B2 phylogenetic group from the A, B1 and D phylogenetic groups in this species. This confirms the classical characteristics of esterases as excellent markers in the study of population genetics for prokaryotes, particularly *Enterobacteriaceae *[32], and eukaryotes such as *Drosophila spp *[33]. Findings from an *in vivo *experimental model of septicaemia did not show direct involvement of Aes in extraintestinal virulence. Moreover, we did not find any virulence-associated genes in the chromosomal region surrounding *aes*. Thus, esterase B does not appear to play a direct role as a virulence factor in *E. coli *extraintestinal infection, but may serve as an informative marker of phylogeny.

## Methods

### Bacterial strains

We used *E*. *coli *K-12 MG1655 (phylogenetic group A) and CFT073 (phylogenetic group B2) reference strains, their mutants, K-12 Δ*aes *(obtained from the KEIO collection [34]) and CFT073 Δ*aes *(obtained during the course of this study) and the *aes *complemented mutant strains K-12 Δ*aes *pACS2 [28] andCFT073 Δ*aes *pACS2 for the identification of the esterase B-encoding gene. The strains K-12 MG1655, CFT073 and their *aes *mutants were also used for the investigation of the putative role of esterase B. We used the 72 strains from the *E. coli *reference (ECOR) collection, encompassing commensal and pathogenic strains representative of the genetic diversity of the species [35], and four additional pathogenic reference strains (536, UTI89, Sakaï and EDL 933) for the sequencing of *aes*. The *E. fergusonii *strain ATCC 35469^T^, the most closely related species to *E. coli *[36], was used as an outgroup.

### Candidate gene selection using bioinformatic tools

The MaGe (Magnifying Genome) software program [14] was used for candidate gene selection and comparative analysis of genetic sequences surrounding *aes*. The MaGe software program allows gene annotation and comparative analysis of available *E. coli *and closely related genomes, with visualisation of *E. coli *genome annotations enhanced by a synchronized display of synteny groups in the other genomes chosen for comparison [14]. Protein motifs and domains can be identified using the InterPro databank [37]. Candidate genes were obtained after the selection of proteins showing esterase motifs and compatible molecular weights (from 15,000 to 60,000 Da) and pI values (from 4.0 to 5.5) [9].

### Inactivation of the *aes *gene and control experiments

Inactivation was carried out as previously described [38], using a PCR product obtained with primers *aes*W1 (5'-TTTCATGGCAGTGGTTCCTTACAATGACGTAATTTG AAAGGAGTTTTTGCGTTAGGCTGGAGCTGCTTC-3') and *aes*W2 (5'-GCCACGCCG GAACATATCGAAATGATGGCTAATCTTGTTGCCGCGTATCGCATATGAAATATCCTCCTTAG-3'). The PCR product contained (i) the FRT-flanked chloramphenicol acetyltransferase (*cat*) gene responsible for chloramphenicol resistance and (ii) the adjacent sequences homologous to the 5' and 3' flanking regions of *aes*. Inactivation of the *aes *gene was confirmed by PCR using the following primers: *aes*1 (5'-ACTGAGCGGCGAATGTTAACA-3') and *aes*2 (5'-ATTGTCTGGAGACGCTGGAA-3'), targeting sequences upstream and downstream from the *aes *gene, respectively; and c1 (5'-TTATACGCAAGGCGACAAGG-3') and c2 (5'-GATCTTCCGTCACAGGTAGG-3'), targeting sequences within *cat *gene. The antibiotic resistance gene was removed using the pCP20 plasmid [38].

Complementation analysis of the mutant strains was carried out by electroporation of the multicopy plasmid pACS2 [28] containing the *aes *gene under its native promoter.

The esterase B phenotype was investigated by vertical slab polyacrylamide gel electrophoresis of crude extracts of parent type, mutant and complemented mutant strains, using 12% (w/v) acrylamide and discontinous Tris/glycine buffer, pH 8.7. Esterase activity was detected by testing for the hydrolysis of 1-naphtyl acetate, as previously described [39].

### Nucleotide sequencing, sequence alignments and selection tests

The *aes *gene was amplified by PCR, using the primers *aes*1 and *aes*2 (see above). The resulting 1250 bp PCR product was then sequenced by the Sanger method [40]. We compared *aes *sequences of 894 bp by sequence alignment using the ClustalW program [41]. The 72 *aes *sequences of the ECOR strains have GenBank accession numbers GQ167069 to GQ167140.

Amino-acid sequences deduced from the nucleotide sequences of *aes *were also analysed. After the generation of the maximum likelihood tree (see below), amino-acid substitutions for each branch of the Aes tree were identified by comparison of consensus sequences between different branches using the SEAVIEW program [42].

We tested for selection with code ML, implemented in PAML [43,44]. Using a maximum likelihood algorithm, PAML assigns likelihood scores to the data according to the various models of selection. Assignment of a higher likelihood score to a model incorporating selection than to a null model without selection and a significative likelihood ratio test are indicative of selection. The overall Ka/Ks ratio (or ω, dN/dS), reflecting selective pressure on a protein-encoding gene, was estimated using the M0 model (one-ratio) [45] for all isolate sequences, with the *E. fergusonii *sequence as an outgroup. We also used the M1a (null) and M2a (positive selection) models [46,47] and the more powerful M7 and M8 models [46,48] to detect positive selection on specific codons (sites). We used the branch-site model A [47,49] for the B2/non-B2 partition. This model is based on the hypothesis that positive selection occurs only in certain branches/lineages.

### Tree reconstruction

Maximum-likelihood phylogenetic trees were all reconstructed using the PHYML program [50] and the GTR+G+I model. This general model is not necessarily the most parsimonious one. However, we also wanted to obtain the bootstrap support values for each partition. Given that (i) the most parsimonious model may differ from one bootstrap resampling to another, and (ii) a very long computer processing time would be required to choose the best model among the 88 possible models for each of the 500 resamplings, we chose a less time-consuming strategy, simply selecting the most general model (GTR+G+I) for all resamplings. We checked that the trees remain the same for the different models, whether the most parsimonious or the most general model is used. Additional file 2: Table S2 gives the different parsimonious models, and their estimated parameters, selected by the Akaike criterion (jMODELTEST version 0.1.1, written by Posada [51], available at http://darwin.uvigo.es/software/jmodeltest.html).

### Tree comparisons

We compared the phylogenetic history of *aes *to the phylogenetic history of the strains, based on the concatenated nucleotide sequences of six housekeeping genes (*trpA*, *trpB*, *pabB*, *putP*, *icd *and *polB*) and individual gene sequences, as described elsewhere [19]. Briefly, each phylogenetic tree *T*_*i *_is firstly transformed into a tree-distance matrix *D*_*i*_, the distance between two strains being the number of branches with positive length connecting them along the tree. The resulting tree distance matrix *D*_*i *_allows the initial tree structure *T*_*i *_to be recovered, independently of branch length. Two tree distance matrices (*D*_*i *_and *D*_*j*_) (corresponding to two gene trees *i *and *j*) can be compared by calculating the Euclidian distance between them (*δ*_*ij*_) [52]. A low *δ*_*ij *_value means that the similarity between the two tree distance matrices *D*_*i *_and *D*_*j *_is high, and, consequently, that their tree structures *T*_*i *_and *T*_*j *_are close. As several gene tree structures are compared through this Euclidian distance metric, a new distance matrix ***Δ ***can be built with the *δ*_*ij *_elements. This ***Δ ***matrix can then be transformed into a "tree of gene trees" using a neighbour-joining algorithm [53].

To obtain a support value for each partition of this tree, we applied this same procedure to 500 bootstrapped sets of data, obtaining 500 ***Δ ***matrices and finally, a bootstrapped consensus "tree of gene trees". A high bootstrap support value separating two sets of gene trees allows incongruent sets of gene trees to be identified; however, a low bootstrap value suggests that the two sets of trees are not incongruent or that there is insufficient phylogenetic information to reject the hypothesis of incongruence. The "TreeOfTree" package is available from the website http://bioinformatics.lif.univ-mrs.fr.

### Protein structure modelling and analysis

Modelling of the Aes protein structure was based on comparison of the available models from MODBASE [54] with models previously obtained using the Tasser-Lite homology modelling server [55,56]. Although some differences were observed between the models obtained by these two independent approaches, in particular in the N terminus region, the best models proposed by Tasser-Lite and MODBASE were similar overall. Given that our aim was to determine only the approximate location of the Aes polymorphism within the protein structure, the MODBASE model was used for further analysis. The model was finally tested to ensure that it contains an active site consistent with esterase activity. This was carried out using the 3D MSS-Sites program http://bioserv.rpbs.jussieu.fr/[57] and the Catalytic Site Atlas [58]. Polymorphic sites were identified by sequence alignment using ClustalW [41] for B_1 _and B_2 _variants separately.

Theoritical pIs of Aes were calculated using the program compute pI of the ExPASY home page http://www.expasy.ch/tools/pi_tool.html.

### *In vitro *growth studies

Competition studies of parent strains K-12 and CFT073, with their respective mutants K-12 Δ*aes*:Kan and CFT073 Δ*aes*:Cm (1/1 ratio), were performed in Luria Bertani (LB) and gluconate minimum liquid media. Gluconate minimal medium mimics the intestinal environment [59]. For each medium and for each competition experiment, bacteria were plated on media with or without the appropriate antibiotic and counted after 2 h (exponential phase) and 18 h (stationary phase). Each experiment was repeated twice. Biolog GN2 (Biolog, Inc., Hayward, CA) plates were used to detect carbon utilisation of 95 substrates. Utilisation of various C sources is coupled to the reduction of a tetrazolium dye and generation of a purple colour [60]. Each strain was grown in LB medium, washed and resuspended to an optical density of 0.01 at 600 nm in mineral medium [60]. Plates were incubated at 37°C and colour changes were measured by changes in optical density (measured on a Tecan microplate reader) at a wavelength of 600 nm. The cut-off for positive results was an optical density of 0.2.

### Septicaemia mouse model

A mouse model of systemic infection was used to assess the intrinsic virulence of the strains [11]. For each strain, 10 outbred female swiss OF1 mice (3-4 weeks old, 14-16 g) were challenged with a standardized subcutaneous bacterial inoculum (2 × 10^8 ^CFU of *E. coli*). Mortality was assessed over seven days following the challenge. Assays were performed using the CFT073 strain as a positive control (killing 10/10 mice), the K-12 strain as a negative control (killing 0/10 mice) [61] and the CFT073 Δ*aes *and CFT073 Δ*aes*:Cm mutant strains. Data were analysed using the StatView software to obtain Kaplan-Meyer curves; statistical analysis was carried out using the logrank test, with p values < 0.05 considered as significant.

## Authors' contributions

ML carried out the *in silico *and *in vitro *studies of Aes, participated to the *in vivo *studies of Aes and wrote the manuscript. CH contributed to the *in silico *studies. OC contributed to the sequencing. LG carried out *in vivo *studies. PD carried out tree comparisons. PT carried out the structural analysis of the protein. ED participated in the design of the study and wrote the manuscript. BP was involved in the initial design of the study and wrote the manuscript. All authors read and approved the final manuscript.

## Authors' Information

ML and CH are PhD students, OC is a research engineer, LG is a technician. PD, PT, ED and BP are researchers.

## Supplementary Material

Additional file 1 **Supplemental figures**. A figure showing the electrophoretic patterns of esterases from various *E. coli *strains. Fig. S1: Polyacrylamide gel electrophoresis of Aes. Gels were stained using 1-naphtyl acetate hydrolysis to detect esterase activity. Esterases B was detected in strains. K-12 (lane 1) and K-12 Δ*aes *pACS2 (lane 3), but not in strain K-12 Δ*aes *(lane 2), thus confirming that *aes *encodes esterase B. The dilution factor used for the crude extract of the complemented strain K-12 Δ*aes *pACS2 was 40 times greater than that of the parent and mutant strains due to overexpression of the *aes *gene on the plasmid. This did not allow us to detect esterase A in the complemented strain, whereas it was clearly visible for the K-12 and K-12 Δ*aes *strains. Fig. S2: Kaplan-Meyer curves showing the comparative scores of virulence in the mouse model of septicaemia as a function of the presence or absence of Aes in the K-12 strain (blue line), CFT073 strain (green line and squares), CFT073 Δ*aes*:Cm strain (red line and circles) and CFT073 Δ*aes *strain (violet line and triangles). Mice inoculated with K-12 strain were still alive at day 7. Click here for file

Additional file 2 **Supplemental Tables**. A table describing the genes surrounding the *aes *gene. Table S1: List of genes of the strain CFT073 and their characteristics within a total region of 150 kbp surrounding the *aes *gene. The *aes *gene and its characteristics are highlighted in red. Table S2: Parsimonious models, and their estimated parameters, selected by the Akaike criterion (jMODELTEST version 0.1.1, written by Posada, 2008, available at http://darwin.uvigo.es/software/jmodeltest.html) used for each tree reconstruction. Click here for file
