# Supplementary material for: aes, the gene encoding the esterase B in Escherichia coli, is a powerful phylogenetic marker of the species
Source: BMC Microbiol. 2009 Dec 29;9:273. doi: 10.1186/1471-2180-9-273 (PMC2805673; doi:10.1186/1471-2180-9-273)
Supplement: Additional file 1 — Supplemental figures. A figure showing the electrophoretic patterns of esterases from various E. coli strains. Fig. S1: Polyacrylamide gel electrophoresis of Aes. Gels were stained using 1-naphtyl acetate hydrolysis to detect esterase activity. Esterases B was detected in strains. K-12 (lane 1) and K-12 Δaes pACS2 (lane 3), but not in strain K-12 Δaes (lane 2), thus confirming that aes encodes esterase B. The dilution factor used for the crude extract of the complemented strain K-12 Δaes pACS2 was 40 times greater than that of the parent and mutant strains due to overexpression of the aes gene on the plasmid. This did not allow us to detect esterase A in the complemented strain, whereas it was clearly visible for the K-12 and K-12 Δaes strains. Fig. S2: Kaplan-Meyer curves showing the comparative scores of virulence in the mouse model of septicaemia as a function of the presence or absence of Aes in the K-12 strain (blue line), CFT073 strain (green line and squares), CFT073 Δaes:Cm strain (red line and circles) and CFT073 Δaes strain (violet line and triangles). Mice inoculated with K-12 strain were still alive at day 7. [file 1471-2180-9-273-S1.PPT]

## Slide 1
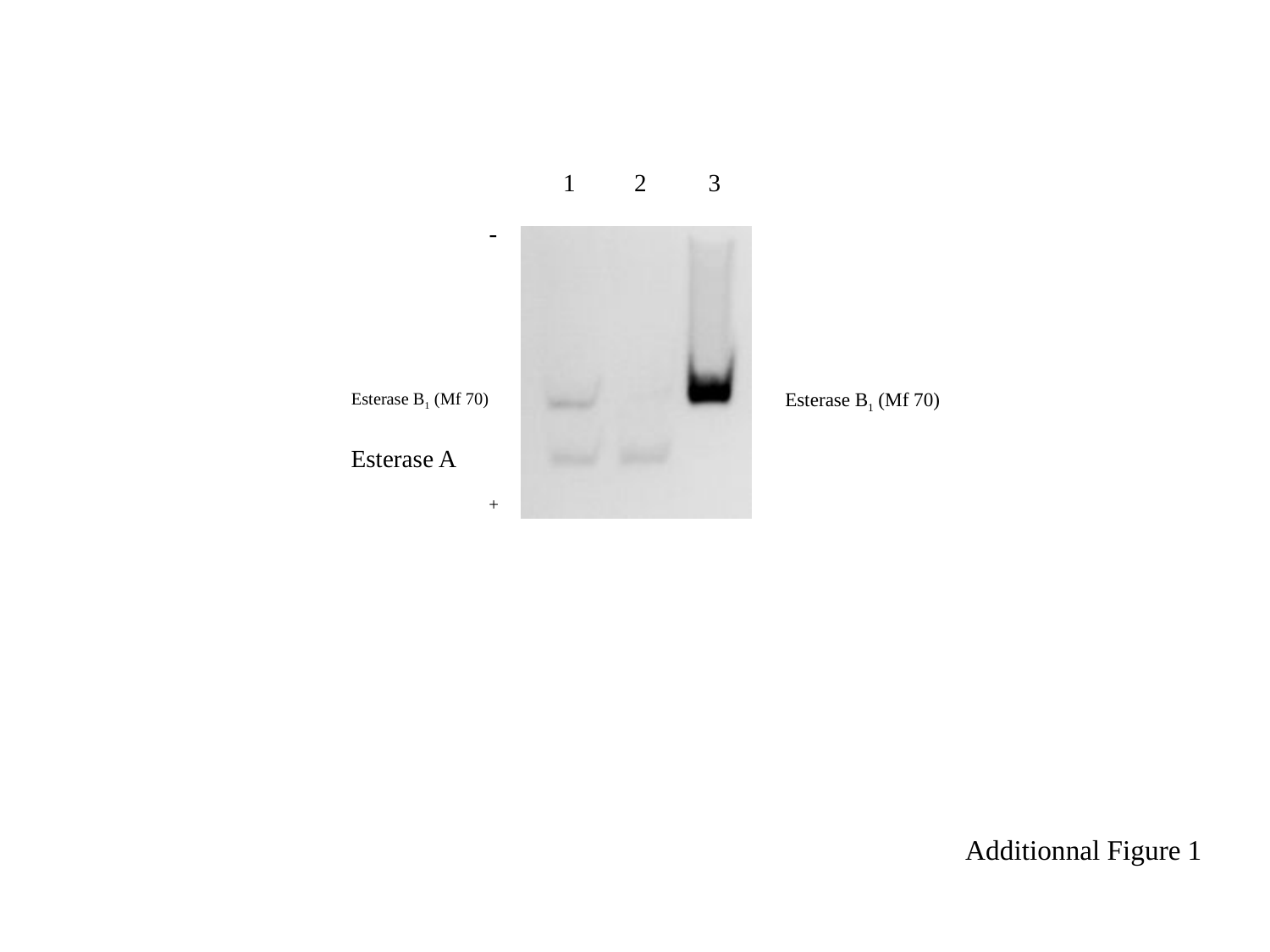

1
2
3
-
Esterase B1 (Mf 70)
Esterase B1 (Mf 70)
Esterase A
+
Additionnal Figure 1

## Slide 2
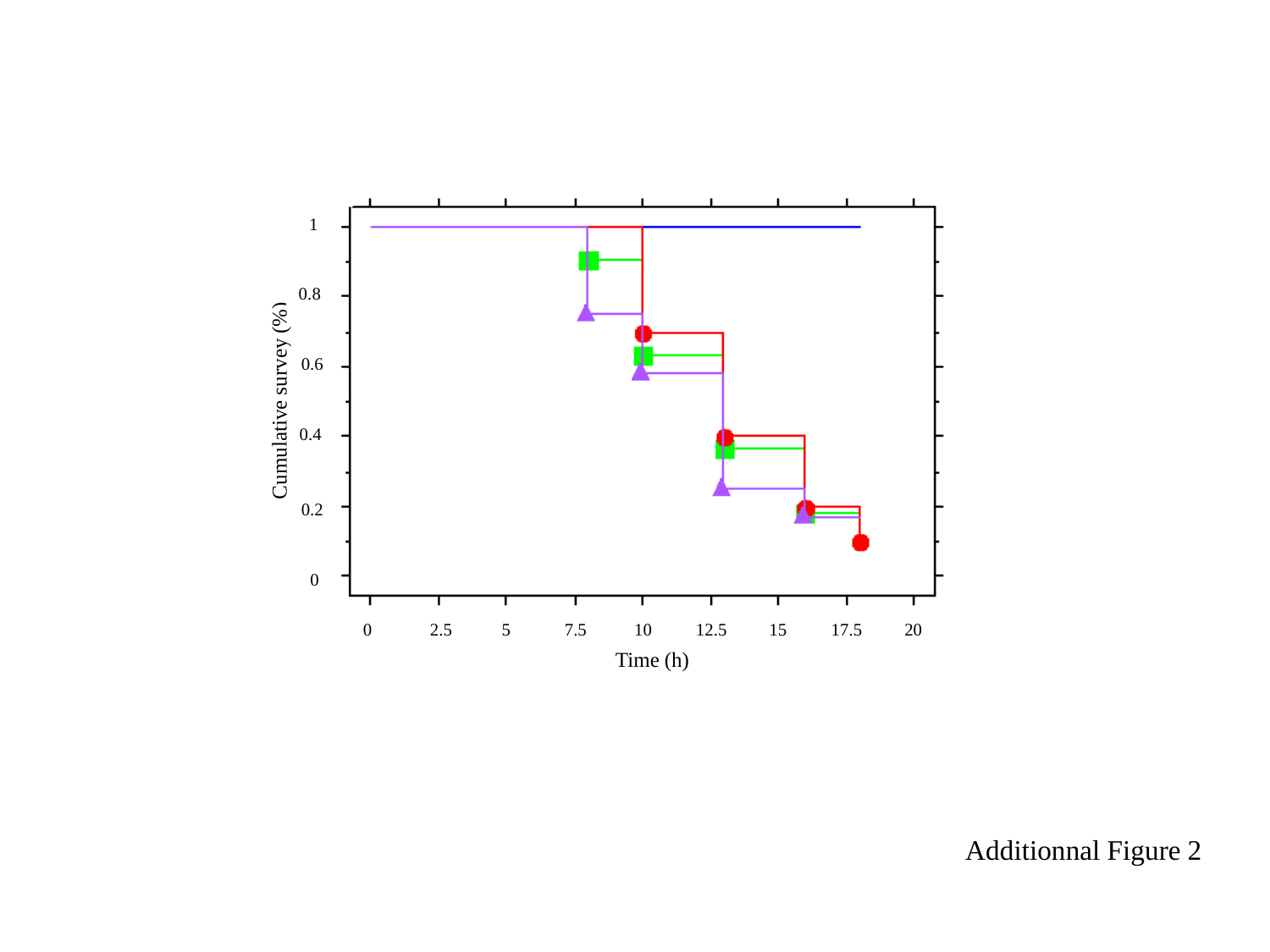

Cumulative survey (%)
1
0.8
0.6
0.4
0.2
0
0
2.5
5
7.5
10
12.5
15
17.5
20
Time (h)
Additionnal Figure 2
