# Supplementary material for: aes, the gene encoding the esterase B in Escherichia coli, is a powerful phylogenetic marker of the species
Source: BMC Microbiol. 2009 Dec 29;9:273. doi: 10.1186/1471-2180-9-273 (PMC2805673; doi:10.1186/1471-2180-9-273)
Supplement: Additional file 2 — Supplemental Tables. A table describing the genes surrounding the aes gene. Table S1: List of genes of the strain CFT073 and their characteristics within a total region of 150 kbp surrounding the aes gene. The aes gene and its characteristics are highlighted in red. Table S2: Parsimonious models, and their estimated parameters, selected by the Akaike criterion (jMODELTEST version 0.1.1, written by Posada, 2008, available at http://darwin.uvigo.es/software/jmodeltest.html) used for each tree reconstruction. [file 1471-2180-9-273-S2.DOC]

Table S1: List of genes of the strain CFT073 and their characteristics within a total region of 150 kbp surrounding the *aes* gene. The *aes* gene and its characteristics are highlighted in red.

| Label | Type | Gene | Begin | End | Frame | Product |
| --- | --- | --- | --- | --- | --- | --- |
| c0514 | CDS | *acpH* | 498403 | 499041 | -2 | acyl carrier protein phosphodiesterase |
| c0515 | CDS | *queA* | 499203 | 500273 | 3 | S-adenosylmethionine:tRNA ribosyltransferase-isomerase |
| c0516 | CDS | *tgt* | 500328 | 501455 | 3 | tRNA-guanine transglycosylase |
| c0517 | CDS | *yajC* | 501448 | 501810 | 1 | SecYEG protein translocase auxillary subunit |
| c0518 | CDS | *secD* | 501838 | 503685 | 1 | SecYEG protein translocase auxillary subunit |
| c0519 | CDS | *secF* | 503651 | 504667 | 2 | SecYEG protein translocase auxillary subunit |
| c0520 | CDS | *yajD* | 504728 | 505144 | 2 | conserved hypothetical protein |
| c0521 | CDS | *tsx* | 505182 | 506114 | -3 | nucleoside channel, receptor of phage T6 and colicin K |
| c0522 | CDS | *yajI* | 506365 | 506964 | -2 | putative lipoprotein |
| c0523 | CDS | *nrdR* | 507055 | 507504 | 1 | transcriptional repressor of nrd genes |
| c0524 | CDS | *ribD* | 507508 | 508611 | 1 | fused diaminohydroxyphosphoribosylaminopyrimidine deaminase;5-amino-6-(5-phosphoribosylamino) uracil reductase |
| c0525 | CDS | *ribE* | 508604 | 509170 | 2 | riboflavin synthase beta chain |
| c0526 | CDS | *_* | 509160 | 509693 | -3 | conserved hypothetical protein |
| c0527 | CDS | *nusB* | 509190 | 509609 | 3 | transcription antitermination protein |
| c0528 | CDS | *thiL* | 509687 | 510664 | 2 | thiamin-monophosphate kinase |
| c0529 | CDS | *pgpA* | 510642 | 511160 | 3 | phosphatidylglycerophosphatase A |
| c0530 | CDS | *yajO* | 511214 | 512188 | -1 | aldoketo-oxidoreductase, NADP-binding |
| c0531 | CDS | *dxs* | 512243 | 514105 | -1 | 1-deoxyxylulose-5-phosphate synthase, thiamine-requiring, FAD-requiring |
| c0532 | CDS | *ispA* | 514130 | 515029 | -1 | geranyltranstransferase |
| c0533 | CDS | *xseB* | 515029 | 515271 | -2 | exonuclease VII small subunit |
| c0534 | CDS | *thiI* | 515477 | 516925 | 2 | sulfurtransferase required for thiamine and 4-thiouridine biosynthesis |
| c0535 | CDS | *yajL* | 516979 | 517575 | -2 | conserved hypothetical protein |
| c0536 | CDS | *panE* | 517532 | 518443 | -1 | 2-dehydropantoate reductase, NADPH-specific |
| c0537 | CDS | *_* | 518443 | 519102 | 1 | conserved hypothetical protein |
| c0538 | CDS | *yajR* | 519230 | 520792 | -1 | putative transporter, major facilitator family |
| c0539 | CDS | *cyoE* | 520743 | 521633 | -3 | protoheme IX farnesyltransferase |
| c0540 | CDS | *cyoD* | 521645 | 521974 | -1 | cytochrome o ubiquinol oxidase subunit IV |
| c0541 | CDS | *cyoC* | 521974 | 522588 | -2 | cytochrome o ubiquinol oxidase subunit III |
| c0542 | CDS | *cyoB* | 522578 | 524569 | -1 | cytochrome o ubiquinol oxidase subunit I |
| c0543 | CDS | *cyoA* | 524591 | 525538 | -1 | cytochrome o ubiquinol oxidase subunit II |
| c0544 | CDS | *ampG* | 525997 | 527472 | -2 | muropeptide transporter |
| c0545 | CDS | *_* | 527471 | 528070 | 2 | conserved hypothetical protein |
| c0546 | CDS | *yajG* | 527516 | 528196 | -1 | putative lipoprotein |
| c0547 | CDS | *_* | 528144 | 528275 | -3 | conserved hypothetical protein |
| c0548 | CDS | *bolA* | 528366 | 528716 | 3 | regulator of penicillin binding proteins and beta lactamase transcription (morphogene) |
| c0549 | CDS | *_* | 528643 | 529065 | -2 | conserved hypothetical protein |
| c0550 | CDS | *_* | 528725 | 528913 | 2 | fragment of conserved hypothetical protein (partial) |
| c0551 | CDS | *tig* | 529060 | 530358 | 1 | peptidyl-prolyl cis/trans isomerase (trigger factor) |
| c0552 | CDS | *_* | 529069 | 530370 | -2 | conserved hypothetical protein |
| c0553 | CDS | *clpP* | 530604 | 531227 | 3 | proteolytic subunit of ClpA-ClpP and ClpX-ClpP ATP-dependent serine proteases |
| c0554 | CDS | *clpX* | 531353 | 532627 | 2 | ATPase and specificity subunit of ClpX-ClpP ATP-dependent serine protease |
| c0555 | CDS | *lon* | 532770 | 535169 | 3 | DNA-binding ATP-dependent protease La |
| c0556 | CDS | *hupB* | 535378 | 535650 | 1 | HU, DNA-binding transcriptional regulator, beta subunit |
| c0557 | CDS | *ppiD* | 535842 | 537713 | 3 | peptidyl-prolyl cis-trans isomerase (rotamase D) |
| c0558 | CDS | *ybaV* | 537864 | 538235 | 3 | conserved hypothetical protein |
| c0559 | CDS | *ybaW* | 538341 | 538739 | 3 | conserved hypothetical protein |
| c0560 | CDS | *queC* | 538791 | 539486 | -3 | queuosine biosynthesis protein |
| c0561 | CDS | *ybaE* | 539551 | 541266 | -2 | conserved hypothetical protein |
| c0562 | CDS | *cof* | 541339 | 542169 | 1 | thiamin pyrimidine pyrophosphate hydrolase |
| c0563 | CDS | *_* | 542302 | 542811 | 1 | transposase IS605 family, IS200 group |
| c0564 | CDS | *ybaO* | 542925 | 543491 | 3 | putative DNA-binding transcriptional regulator with homology to Lrp |
| c0565 | CDS | *mdlA* | 543521 | 545293 | 2 | putative fused ATPase and permease component of metabolite transporter |
| c0566 | CDS | *mdlB* | 545172 | 547067 | 3 | putative fused ATPase and permease component of metabolite ABC transporter |
| c0567 | CDS | *_* | 547081 | 547221 | -2 | conserved hypothetical protein |
| c0568 | CDS | *glnK* | 547248 | 547586 | 3 | nitrogen assimilation regulatory protein for GlnL, GlnE, and AmtB |
| c0569 | CDS | *_* | 547305 | 547538 | -3 | conserved hypothetical protein |
| c0570 | CDS | *amtB* | 547616 | 548902 | 2 | ammonium transporter |
| c0571 | CDS | *tesB* | 548951 | 549895 | -1 | acyl-CoA thioesterase II |
| c0572 | CDS | *ybaY* | 550029 | 550601 | 3 | putative lipoprotein |
| c0573 | CDS | *ybaZ* | 550632 | 551042 | -3 | putative methylated DNA-protein cysteine alkyltransferase |
| c0574 | CDS | *ybaA* | 551322 | 551675 | 3 | conserved hypothetical protein |
| c0575 | CDS | *ylaB* | 551717 | 553273 | -1 | conserved hypothetical protein; putative inner membrane protein |
| c0576 | CDS | *ylaC* | 553431 | 553940 | -3 | conserved hypothetical protein; putative inner membrane protein |
| c0577 | CDS | *maa* | 554017 | 554568 | -2 | maltose O-acetyltransferase |
| c0578 | CDS | *hha* | 554741 | 554971 | -1 | modulator of gene expression, with H-NS |
| c0579 | CDS | *ybaJ* | 554985 | 555359 | -3 | conserved hypothetical protein |
| c0580 | CDS | *acrB* | 555904 | 559053 | -2 | multidrug efflux system protein |
| c0581 | CDS | *acrA* | 559076 | 560305 | -1 | multidrug efflux system |
| c0582 | CDS | *acrR* | 560411 | 561058 | 2 | DNA-binding transcriptional repressor |
| c0583 | CDS | *_* | 561140 | 561304 | 2 | conserved hypothetical protein |
| c0584 | CDS | *kefA* | 561186 | 564548 | 3 | fused conserved hypothetical protein ; mechanosensitive channel protein |
| c0585 | CDS | *ybaM* | 564760 | 564921 | -2 | conserved hypothetical protein |
| c0586 | CDS | *priC* | 564935 | 565462 | -1 | primosomal replication protein N'' |
| c0587 | CDS | *ybaN* | 565532 | 565909 | 2 | conserved hypothetical protein; putative inner membrane protein |
| c0588 | CDS | *apt* | 566008 | 566613 | 1 | adenine phosphoribosyltransferase |
| c0589 | CDS | *dnaX* | 566742 | 568673 | 3 | DNA polymerase III/DNA elongation factor III, tau and gamma subunits |
| c0590 | CDS | *_* | 568702 | 569079 | -2 | conserved hypothetical protein |
| c0591 | CDS | *ybaB* | 568711 | 569055 | 1 | conserved hypothetical protein |
| c0592 | CDS | *recR* | 569055 | 569660 | 3 | gap repair protein |
| c0593 | CDS | *htpG* | 569770 | 571644 | 1 | molecular chaperone HSP90 family |
| c0594 | CDS | *adk* | 571765 | 572469 | 1 | adenylate kinase |
| c0595 | CDS | *hemH* | 572601 | 573563 | 3 | ferrochelatase |
| c0596 | CDS | *aes* | 573560 | 574519 | -1 | acetyl esterase |
| c0597 | CDS | *gsk* | 574671 | 575975 | 3 | inosine/guanosine kinase |
| c0598 | CDS | *ybaL* | 576105 | 577781 | -3 | putative monovalent cation:proton antiporter (CPA2 family) |
| c0599 | CDS | *fsr* | 578019 | 579239 | -3 | fosmidomycin efflux system, member of the major facilitator superfamily |
| ECOLI0561 | CDS | *_* | 579275 | 579460 | 2 | conserved hypothetical protein |
| c0600 | CDS | *ushA* | 579457 | 581109 | 1 | bifunctional UDP-sugar hydrolase and 5'-nucleotidase |
| c0601 | CDS | *ybaK* | 581146 | 581625 | -2 | conserved hypothetical protein |
| c0602 | CDS | *ybaP* | 581829 | 582623 | -3 | conserved hypothetical protein |
| ECOLI0565 | CDS | *_* | 582872 | 583057 | 2 | fragment of putative RelE/ParE family protein, cytotoxic translational repressor of toxin- antitoxin stability system (partial) |
| c0603 | CDS | *ybaQ* | 583093 | 583434 | 1 | putative DNA-binding transcriptional regulator |
| c0604 | CDS | *copA* | 583492 | 585996 | -2 | copper transporter |
| c0605 | CDS | *ybaS* | 586259 | 587191 | 2 | amidase, possibly glutaminase |
| c0606 | CDS | *ybaT* | 587194 | 588486 | 1 | putative nitrogen-containing metabolite transporter |
| c0607 | CDS | *cueR* | 588611 | 589018 | 2 | DNA-binding transcriptional activator of copper-responsive regulon genes |
| c0608 | CDS | *_* | 589019 | 590242 | -1 | conserved hypothetical protein |
| c0609 | CDS | *ybbJ* | 590361 | 590819 | -3 | conserved hypothetical protein; putative inner membrane protein |
| c0610 | CDS | *ybbK* | 590816 | 591733 | -1 | putative protease, membrane anchored |
| c0611 | CDS | *ybbL* | 591876 | 592556 | 3 | putative transporter subunit: ATP-binding component of ABC superfamily |
| c0612 | fCDS | *ybbM* | 592516 | 592800 | 1 | fragment of putative permease of an ABC transporter (part 1) |
| c0612 | fCDS | *ybbM* | 592769 | 593323 | 2 | fragment of putative permease of an ABC transporter (part 2) |
| c0613 | CDS | *ybbN* | 593386 | 594276 | -2 | putative thioredoxin domain-containing protein |
| c0614 | CDS | *ybbO* | 594301 | 595110 | -2 | putative oxidoreductase with NAD(P)-binding Rossmann-fold domain |
| c0615 | CDS | *tesA* | 595100 | 595756 | -1 | multifunctional acyl-CoA thioesterase I and protease I and lysophospholipase L1 |
| c0616 | CDS | *ybbA* | 595694 | 596380 | 2 | putative transporter subunit: ATP-binding component of ABC superfamily |
| c0617 | CDS | *ybbP* | 596377 | 598791 | 1 | putative ABC transporter permease |
| c0618 | CDS | *ybbB* | 598942 | 599901 | -2 | tRNA 2-selenouridine synthase, selenophosphate-dependent |
| c0619 | CDS | *ybbS* | 600096 | 601022 | -3 | putative DNA-binding transcriptional activator of the allD operon |
| c0620 | CDS | *allA* | 601252 | 601734 | 1 | ureidoglycolate hydrolase |
| c0621 | CDS | *allR* | 601812 | 602627 | 3 | DNA-binding transcriptional repressor |
| c0622 | CDS | *gcl* | 602627 | 604498 | 2 | glyoxylate carboligase |
| c0623 | CDS | *hyi* | 604511 | 605287 | 2 | hydroxypyruvate isomerase |
| c0624 | CDS | *glxR* | 605387 | 606265 | 2 | tartronate semialdehyde reductase, NADH-dependent |
| c0625 | CDS | *ybbW* | 606435 | 607889 | 3 | allantoin or uracil transporter |
| c0626 | CDS | *allB* | 607949 | 609310 | 2 | allantoinase |
| c0627 | CDS | *ybbY* | 609360 | 610667 | 3 | putative uracil/xanthine transporter |
| c0628 | CDS | *glxK* | 610683 | 611834 | 3 | glycerate kinase II |
| c0629 | CDS | *ylbA* | 611963 | 612748 | -1 | conserved hypothetical protein |
| c0630 | CDS | *allC* | 612759 | 614012 | -3 | allantoate amidohydrolase |
| c0631 | CDS | *allD* | 614016 | 615065 | -3 | ureidoglycolate dehydrogenase |
| c0632 | CDS | *fdrA* | 615382 | 617049 | 1 | putative acyl-CoA synthetase with NAD(P)-binding Rossmann-fold domain |
| c0633 | CDS | *ylbE* | 617059 | 618318 | 1 | conserved hypothetical protein |
| c0634 | CDS | *ylbF* | 618236 | 619144 | 2 | conserved hypothetical protein |
| c0635 | CDS | *ybcF* | 619141 | 620034 | 1 | putative carbamate kinase |
| c0636 | CDS | *purK* | 620167 | 621234 | -2 | N5-carboxyaminoimidazole ribonucleotide synthase |
| c0637 | CDS | *purE* | 621231 | 621767 | -3 | N5-carboxyaminoimidazole ribonucleotide mutase |
| c0638 | CDS | *_* | 621836 | 622165 | -1 | putative membrane protein |
| c0639 | CDS | *lpxH* | 622276 | 622998 | -2 | UDP-2,3-diacylglucosamine pyrophosphatase |
| c0640 | CDS | *_* | 622989 | 623675 | 3 | conserved hypothetical protein |
| c0641 | CDS | *ppiB* | 623001 | 623495 | -3 | peptidyl-prolyl cis-trans isomerase B (rotamase B) |
| c0642 | CDS | *cysS* | 623669 | 625054 | 2 | cysteinyl-tRNA synthetase |
| c0643 | CDS | *_* | 625090 | 625527 | -2 | conserved hypothetical protein |
| c0644 | CDS | *ybcJ* | 625720 | 625953 | -2 | putative RNA-binding protein |
| c0645 | CDS | *folD* | 625934 | 626800 | -1 | bifunctional 5,10-methylene-tetrahydrofolate dehydrogenase and 5,10-methylene-tetrahydrofolate cyclohydrolase |
| c0646 | CDS | *_* | 626841 | 627038 | 3 | conserved hypothetical protein |
| c5517 | tRNA | *_* | 627071 | 627147 | 1 | tRNA-Arg |
| c0647 | fCDS | *intD* | 627155 | 627535 | -1 | fragment of putative integrase; DLP12 prophage (part 2) |
| c0648 | fCDS | *intD* | 627483 | 627782 | -3 | fragment of putative integrase; DLP12 prophage (part 1) |
| c0649 | CDS | *tfaQ* | 627789 | 628145 | 3 | Tail fiber assembly protein |
| c0650 | CDS | *_* | 628118 | 628243 | 2 | conserved hypothetical protein |
| c0651 | CDS | *ybcY* | 628200 | 628865 | -3 | putative AdoMet-dependent methyltransferase; DLP12 prophage |
| c0652 | CDS | *ompT* | 629100 | 630053 | -3 | outer membrane protease VII (outer membrane protein 3b); DLP12 prophage |
| ECOLI0613 | CDS | *_* | 630457 | 630606 | -2 | hypothetical protein |
| c0653 | CDS | *ybcH* | 630712 | 631602 | -2 | conserved hypothetical protein |
| c0654 | CDS | *nfrA* | 631603 | 634596 | -2 | bacteriophage N4 receptor, outer membrane subunit |
| c0655 | CDS | *nfrB* | 634562 | 636724 | -1 | bacteriophage N4 receptor, inner membrane subunit |
| c0656 | CDS | *cusS* | 636949 | 638391 | -2 | sensory histidine kinase in two-component regulatory system with CusR, senses copper ions |
| c0657 | CDS | *cusR* | 638381 | 639064 | -1 | DNA-binding response regulator in two-component regulatory system with CusS |
| c0658 | CDS | *cusC* | 639221 | 640603 | 2 | copper/silver efflux system, outer membrane component |
| c0659 | CDS | *cusF* | 640627 | 640959 | 1 | periplasmic copper-binding protein |
| c0660 | CDS | *cusB* | 640975 | 642198 | 1 | copper/silver efflux system, membrane fusion protein |
| c0661 | CDS | *cusA* | 642210 | 645353 | 3 | copper/silver efflux system, membrane component |
| c0662 | CDS | *pheP* | 645419 | 646837 | 2 | phenylalanine transporter |
| c0663 | CDS | *ybdG* | 646994 | 648241 | -1 | putative membrane protein; putative channel |
| c0664 | CDS | *nfnB* | 648349 | 649002 | -2 | dihydropteridine reductase, NAD(P)H-dependent, oxygen-insensitive |

Table S2: Different parsimonious models, and their estimated parameters, selected by the Akaike criterion (jMODELTEST version 0.1.1, written by Posada, 2008, available at <http://darwin.uvigo.es/software/jmodeltest.html>) used for each tree reconstruction.

|  | *aes* | *icd* | *pabB* | *polB* | *putP* | *trpA* | *trpB* | MLST |
| --- | --- | --- | --- | --- | --- | --- | --- | --- |
|  |  |  |  |  |  |  |  |  |
| Model | SYM+G | TIM1ef+I+G | TVMef+G | TIM1+G | TrN+I+G | K80+G | GTR+G | GTR+I+G |
|  |  |  |  |  |  |  |  |  |
| pA | 0.250 | 0.250 | 0.250 | 0.228 | 0.170 | 0.250 | 0.233 | 0.222 |
| pC | 0.250 | 0.250 | 0.250 | 0.263 | 0.221 | 0.250 | 0.268 | 0.258 |
| pG | 0.250 | 0.250 | 0.250 | 0.289 | 0.339 | 0.250 | 0.288 | 0.281 |
| pT | 0.250 | 0.250 | 0.250 | 0.221 | 0.270 | 0.250 | 0.212 | 0.240 |
|  |  |  |  |  |  |  |  |  |
| AC | 3.334 | 1.000 | 1.862 | 1.000 | 1.000 | k=5.143 | 0.343 | 0.671 |
| AG | 6.863 | 3.272 | 8.365 | 12.101 | 4.044 |  | 1.877 | 4.600 |
| AT | 1.034 | 2.570 | 3.866 | 1.803 | 1.000 |  | 1.004 | 1.611 |
| CG | 0.771 | 2.570 | 2.405 | 1.803 | 1.000 |  | 0.927 | 1.090 |
| CT | 11.542 | 10.247 | 8.365 | 8.369 | 7.094 |  | 3.385 | 6.019 |
| GT | [1] | [1] | [1] | [1] | [1] |  | [1] | [1] |
| p-Inv |  | 0.669 |  |  | 0.415 |  |  | 0.574 |
| G (8classes) | 0.213 | 0.401 | 0.199 | 0.094 | 0.292 |  | 0.258 | 0.317 |

Substitution models including invariable sites (+I) and the rate of variation among sites (+G), or both (+I+G).

SYM+G: f(A)=f(T)=f(C)=f(G); ACAGATCGCT; G

TIM1ef+I+G: f(A)=f(T)=f(C)=f(G); AC=GTAT=CGAG, CT; I; G

TVMef+G: f(A)=f(T)=f(C)=f(G); ACATCGGTAG=CT; G

TIM1+G: f(A)≠f(T)≠f(A)≠f(T)≠f(C)≠f(G); AC=GTAT=CGAGCT; G

TrN+I+G: f(A)≠f(T)≠f(A)≠f(T)≠f(C)≠f(G); AC=AT=CG=GTAGCT; I;G

K80+G: f(A)=f(T)=f(C)=f(G); AC=AT=CG=GT AG=CT; G

GTR+G: f(A)≠f(T)≠f(A)≠f(T)≠f(C)≠f(G); ACAGATCGCT; G

GTR+I +G: f(A)≠f(T)≠f(A)≠f(T)≠f(C)≠f(G); ACAGATCGCT; I; G
